# Age-Associated Changes of Nasal Bacterial Microbiome in Patients With Chronic Rhinosinusitis

**DOI:** 10.3389/fcimb.2022.786481

**Published:** 2022-02-17

**Authors:** Fang Chen, Wenxiang Gao, Chaosheng Yu, Junzheng Li, Feng Yu, Meng Xia, Jiajian Liang, Jianbo Shi, Yinyan Lai

**Affiliations:** ^1^ Department of Otorhinolaryngology-Head and Neck Surgery, Guangzhou Red Cross Hospital, Jinan University, Guangzhou, China; ^2^ Department of Otorhinolaryngology, First Affiliated Hospital of Sun Yat-sen University, Sun Yat-sen University, Guangzhou, China; ^3^ Guangzhou Medical University, Guangzhou, China

**Keywords:** chronic rhinosinusitis, microbiota, 16S rDNA, bacterial composition, predicted functional profiles, metabolic pathway

## Abstract

Age-related changes in nasal bacterial microbiota of patients with chronic rhinosinusitis (CRS) remains unclear. In this study, we aimed to identify distinct characteristics of nasal bacterial microbiota between aged and younger patients with CRS through 16S rDNA gene sequencing. Patients with CRS undergoing endoscopic sinus surgery were recruited and separated into aged (≥60 years, median age = 66 years, N = 17) and younger (<60 years, median age = 35.5 years, N = 14) patients. Diversity, bacterial composition and metabolic activities of nasal microbiota between aged and younger patients were compared. Results have shown that levels of OTUs (p = 0.0173) and microbiota diversity (all p < 0.05) decreased significantly in aged patients. The abundance of phylum *Actinobacteria*, and genus *Corynebacterium* were significantly higher in aged patients, while the abundance of phylum *Bacteroidetes*, *Fusobacteria*, and genus *Fusobacterium*, *Peptoniphilus* were significantly higher in younger patients. In addition, predicted functional profiles have revealed that 41 KEGG pathways involving in 12 metabolic pathways, 4 genetic information processing, 3 environmental information processing, 4 cellular processes, 8 organismal systems, 6 human diseases, and 4 unclassified pathways were identified. Among which, the vast majority of metabolic activities are involved in replication and repair, membrane transport, translation, and the metabolism of amino acid, carbohydrate, energy, cofactors and vitamins, and nucleotide. On the level of the thirdly bacterial metabolic pathways, purine metabolism, glycine, serine and threonine metabolism, valine, leucine and isoleucine biosynthesis, glycolysis/gluconeogenesis and phenylalanine, tyrosine and tryptophan biosynthesis are significantly up-regulated while carbon fixation pathways in prokaryotesand methane metabolism are significantly down-regulated in aged patients. Overall, our analysis revealed that age-related physiological and pathological changes on the nasal mucosal surface may alter the host immune response and be highly associated with the nasal bacterial microbiota of patients with CRS. However, future studies are needed to elucidate the causal relationship.

## Introduction

Chronic rhinosinusitis (CRS) is a very heterogeneous sinonasal inflammatory disease with persistent symptoms and recurrent exacerbations that affects around 10% of the population world-wide, resulting in a significant socioeconomic burden (DeConde and Soler, 2016; Jang et al., 2021). Despite simple diagnostic criteria are achieved based on clinical symptoms and objective findings (Fokkens et al., 2020), treatment strategies for CRS are limited due to their poorly pathophysiological definition. Previous studies suggest that CRS exhibited age-related changes to the sinonasal tract and epithelium, including decline in epithelial barrier function (Cho et al., 2015), reduced mucociliary clearance (Ho et al., 2001), and decrement in neutrophilic inflammation (Shaw et al., 2013), which potentially increased the incidence of CRS and the severity of symptoms in elderly population. For example, a Korean epidemiological study by Kim et al (Kim et al., 2011) showed that the incidence of CRS in patients after 60 years old was almost doubled compared to young patients between the ages of 19 and 39. Other evidence suggests that aged patients with CRS suffer from more exaggerated neutrophilic proinflammatory response to pathogenic bacteria (Morse et al., 2019), less improvement in quality-of-life outcomes after sinus surgery (Yancey et al., 2019), and higher antibiotic utilization (Ramakrishnan et al., 2017). With a rapidly aging population of China, CRS and other chronic diseases will likely continue to burden the health care economy and drive increase in costs to delivering care. Therefore, it would be critical to elucidate age-associated differences in treatment response and pathophysiology of CRS for cost reduction, quality improvement and individualized treatment.

Numerous aetiologies resulted from complex interplays among environment, host, and microbial factors have been examined to clarify the pathophysiology of CRS (Hayes et al. 2020; Eschenbacher et al., 2021). Recently, disturbance of the nasal microbiome is recognized as one of the factors influencing CRS pathophysiology since a shift in the balance between commensal and potentially pathogenic microorganisms is well-recognized features in patients with CRS (Stevens et al., 2015; Cope et al., 2017; Vickery et al., 2019). For example, Gan et al., (2021) demonstrated that microbial dysbiosis in the nasal cavity is associated with the pathogenesis of chronic rhinosinusitis with nasal polyps (CRSwNP) *via* high-throughput sequencing technology based on 16S rRNA. In addition, the decrease in protective bacteria and the increase in pathogenic bacteria are potentially correlated with the recurrence of nasal polyps. A meta-analysis conducted by Wagner Mackenzie et al. (2017) revealed that levels of specific gatekeeping genera, such as *Corynebacterium*, *Peptoniphilus*, and *Propionobacterium*, have been reduced in the microbiota of CRS patients, which might predispose the host to pathogen-driven inflammation. So far, much effort has been done to define the relationship between the type and amount of the microorganism and the severity of CRS using similar methods in microbiota analysis (Cho et al., 2021; Delliere et al., 2021; Stryjewska-Makuch et al., 2021). Collectively, these studies suggest that bacterial microbiota might play a pathogenic role in CRS, with significant implications for disease management and treatment. Age-dependent factors might be important for nasal bacterial microbiome in patients with CRS due to balanced inter-microbial exchange maintains the microbiota community and leads to anti-inflammatory host interactions. However, to the best of our knowledge, few studies have evaluated the differences in the bacterial community among aged CRS patients compared with younger CRS patients.

In this study, we aimed to identify distinct characteristics of nasal bacterial microbiota between aged and younger patients with CRS, which holds the potential to change treatment approaches for this at-risk population.

## Materials and Methods

### Study Design and Population

This cross-sectional study was approved by the Institutional Review Board of Jinan University Guangzhou Red Cross Hospital (Medical Ethics Trial 2021-064-01) and registered with ChiCTR China Clinical Trials (ChiCTR2100044314). All participants provided written informed consent, and this study was carried out in accordance with the Declaration of Helsinki.

A total of 31 patients with CRS who underwent endoscopic sinus surgery in the Department of Otorhinolaryngology-Head and Neck Surgery of Guangzhou Red Cross Hospital were enrolled in this study. All CRS subjects were diagnosed based on personal history, physical examination, nasal endoscopy, and CT findings of the sinuses according to the 2020 European position paper on rhinosinusitis and nasal polyp guidelines and Nasal Polyps and the International Consensus Statement on Allergy and Rhinology (Orlandi et al., 2016; Fokkens et al., 2020). The exclusion criteria were as follows: (1) Age under 18 years of age, (2) Treatment with antibiotics and systemic steroids less than 4 weeks prior to the ESS, (3) Active infection, (4) Congenital and acquired immunodeficiency, cystic fibrosis. (5) Neoplastic process of the nose and paranasal sinuses, (6) Fungal sinusitis. CRS subjects at 18 years or older and outside the exclusion criteria were divided into aged group (patients aged 60 years or older) and younger group (patients aged 18-59 years) by age to investigate the associations between changes of bacterial community and age.

### Mucus Collection and DNA Extraction

Mucus samples were collected carefully by inserting a Puritan PurFlock swab (Puritan Diagnostics, Guilford, ME) into the right nasal cavity and then passed off the field and placed in a sterile microcentrifuge tube on ice until transport to the -80°C freezer where it was stored until DNA extraction.

Total bacterial genomic DNA extraction was performed using a DNeasy Blood & Tissue Kit (Qiagen, Hilden, Germany) and processed according to manufacturer’s protocol. The DNA concentration was measured using BioSpec-nano (Shimadzu, Japan).

### Amplification and Pyrosequencing of the 16S rDNA Gene

The V3 and V4 variable regions of bacterial 16S ribosomal DNA gene were amplified using KOD PCR Master Mix (Aidlab, Beijing, China) with the forward primer (5’- CCT ACG GRR BGC ASC AGK VRV GAA T-3’) and reverse primer (5’- GGA CTA CNV GGG TWT CTA ATC C-3’). Amplifications were carried out under the following protocols: denatured at 95°C for 3 min, followed by 27 cycles at 95°C for 10 s, 62°C for 30 s, and 72°C for 30 s, with a final extension at 72°C for 10 min. The PCR products were confirmed by 2% agarose gel electrophoresis and purified using the AxyPrep DNA Gel Extraction Kit (Axygen Biosciences, Union City, CA, USA). The PCR products were subsequently quantified using QuantiFluor-ST (Promega, Madison, WI, USA) and sequenced on the Illumina MiSeq PE250 system (Illumina, San Diego, CA, USA) according to the manufacturer’s instructions.

### Date Processing and Bioinformatics Analysis

The raw sequence reads were pre-filtered using QIIME v1.9.1 (Caporaso et al., 2010) to remove sequences with low quality scores, using CUTADAPT [18] to remove adapter sequences and using the FASTX-toolkit[19] to further trim ambiguous bases. Then, paired and clean reads were merged using FLASH (Magoc and Salzberg, 2011) with a minimum overlap of 20 bp and mismatch error rates of 2% and further filtered by QIIME v1.9.1 to obtain the high quality sequencing data. Downstream data analysis was completed using QIIME v1.9.1 with the EzBiocloud 16S rRNA gene sequence database (Edgar, 2010). Operational taxonomic units (OTUs) were defined as clusters of sequences with 97% similarity or higher using mothur v1.39.1 (https://www.mothur.org/) and UPARSE (Edgar, 2013) pipeline. Alpha-diversity indexes, including Chao 1, Shannon index, and Simpson’s index, were analyzed by QIIME v1.9.1 using the core_diversity_analyses option to evaluate bacterial richness and diversity. Beta diversity analysis was performed according to the Bray-Curtis calculation method and visualized using principal co-ordinates analysis (PCoA) to analyze the difference in bacterial community composition between samples. The functional annotation of predicted features was carried out based on the Kyoto Encyclopedia of Genes and Genomes (KEGG) database. Predictive functional profiles of microbial communities were obtained using PICRUSt2 (Douglas et al., 2020) (http://huttenhower.sph.harvard.edu/galaxy/) with default parameters based on the species annotation and abundance of effective OTUs. Based on the prediction results, differences in KEGG pathways related to metabolism and organismal systems between aged and younger patients were calculated and visualized by STAMP v2.1.3 (Parks et al., 2014). Pearson correlation coefficient analyses were conducted to reveal the correlations between the bacterial diversity and bacterial metabolic pathway.

### Statistical Analysis

Demographic data management and analysis was conducted with SPSS 20.0 (IBM, New York, USA). High-throughput sequencing data management and analysis was performed using R software (version 3.4; R Foundation for Statistical Computing, Vienna, Austria) and Prism 5 software (GraphPad, California, USA). Differences in demographics between groups were assessed by using the Kruskal-Wallis test or Chi-square test. Differences in the microbiomes at each level (OUTs, phylum and genus) between aged patients and young patients were confirmed by using the Welch’s t-tests. For all hypothesis tests, results with a p value of less than 0.05 were considered statistically significant.

## Results

### Study Population and Demographics

A total of 31 patients aged 21 to 79 years (median age = 62 years) with CRS who underwent endoscopic sinus surgery were separated into aged (≥ 60 years) and younger (<60 years) patients to investigate the age-related changes in bacterial community of nasal cavity (**Table 1**). The overall severity of disease was significant, with a high frequency of nasal polyps (48%), a mean SNOT-22 score of 42.5 and median disease duration of 44 months. Among which, the aged group consisted of 17 patients aged 63 to 79 years with a median age of 66 years and the younger group consisted of 14 patients aged 21 to 59 years with a median age of 54 years. No significant differences in all demographically assessed variables, such as sex (53% *vs* 64%, p = 0.717), frequency of nasal polyps (41% *vs* 57%, p = 0.479), disease duration (44 *vs* 43, p = 0.897), and SNOT-22 score (41.6 *vs* 43.7, p = 0.587), were found between aged and younger patients.

**Table 1:** Differences in demographic and clinical characteristics between aged and younger patients with CRS.

	Patients with CRS^a^			
	Total	Aged patients	Younger patients	p value
**No.**	31	17	14	
**Age (y)**	62 (21-79)	66 (63-79)	35.5 (21-59)	<0.0001
**Male sex, no. (%)**	18 (58)	9 (53)	9 (64)	0.717
**Nasal polyps, no. (%)**	15 (48)	7 (41)	8 (57)	0.479
**Disease duration (month)**	44 (31-56)	44 (29.5-56)	43 (24-56)	0.897
**SNOT-22 score**	42.5 ± 10.6	41.6 ± 9.0	43.7 ± 12.6	0.587

a Values are presented as either means ± SDs or medians with ranges, depending on the normalcy of the data.

### Decrease in Bacterial Richness and Diversity in Aged Patients With CRS

**Figure 1:**
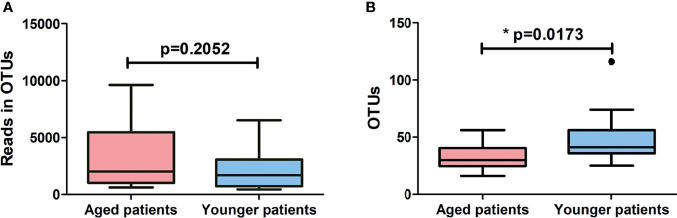
Comparison of **(A)** reads in OTUs and **(B)** number of OTUs identified between aged and younger patients. *p < 0.05.

**Figure 2:**
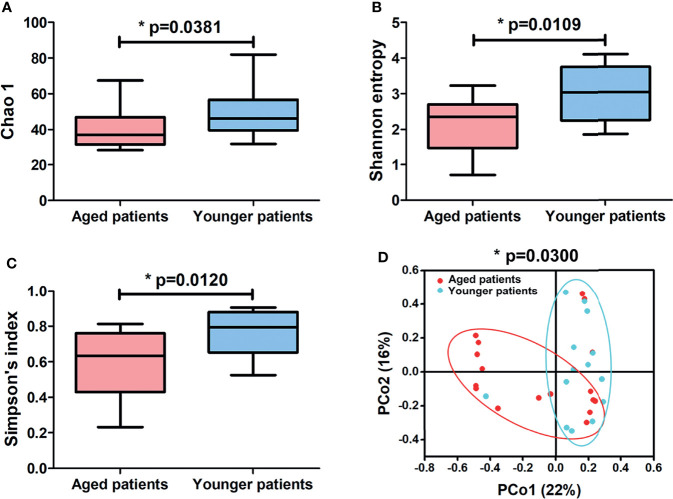
Differences in alpha-diversity indexes and principal coordinate analysis (PCoA) of aged and younger patients. Comparison of **(A)** Chao 1, **(B)** Shannon entropy and **(C)** Simpson’s index between aged and younger patients. **(D)** Principal coordinate analysis plot of bacterial community of aged and younger patients according to Bray-Curtis calculation method. A significant difference in bacterial microbiota (p = 0.0300) between aged and younger patients was shown by permutational multivariate ANOVA (PERMANOVA) on OTUs level according to Bray–Curtis dissimilarity index. *p < 0.05.

A total of 1957981 high quality valid sequences were obtained by merging and filtering raw sequence from each sample, with an average effective ratio of 99.5% and an average sequence length of 248.2 bp after trimming (**Supplementary Table 1**). Among which, a total of 87968 effective sequences were clustered into 427 OTUs using a 97% similarity threshold (**Supplementary Table 1**). Potential relationships between age and bacterial richness and diversity were further investigated by comparing patients of advanced age with their younger counterparts. As shown in **Figure 1**, aged patients with CRS showed similar levels of reads in OTUs (p = 0.2052) and a significant decrease in levels of OTUs (p = 0.0173) compared to younger patients. The alpha-diversity indexes and beta-diversity varied between aged and younger patients are shown in **Figure 2**. The values of Chao 1, Shannon entropy and Simpson’s index in aged patients were significantly lower than the values for younger patients (all p < 0.05, **Figures 2A–C**), indicating that aging has been associated with decreased levels of bacterial richness and diversity in patients with CRS. The differences in bacterial community composition between aged and younger patients were analyzed using principal coordinate analysis (PCoA) based on the relative abundance of OTUs (**Figure 2D**). A relatively clear separation and a significant difference in bacterial microbiota (p = 0.0300) on OTUs level were observed between the aged and younger patients, suggesting that the bacterial community composition changed greatly with aging.

### Differences in Composition of Bacterial Community Between Aged and Younger Patients

**Figure 3:**
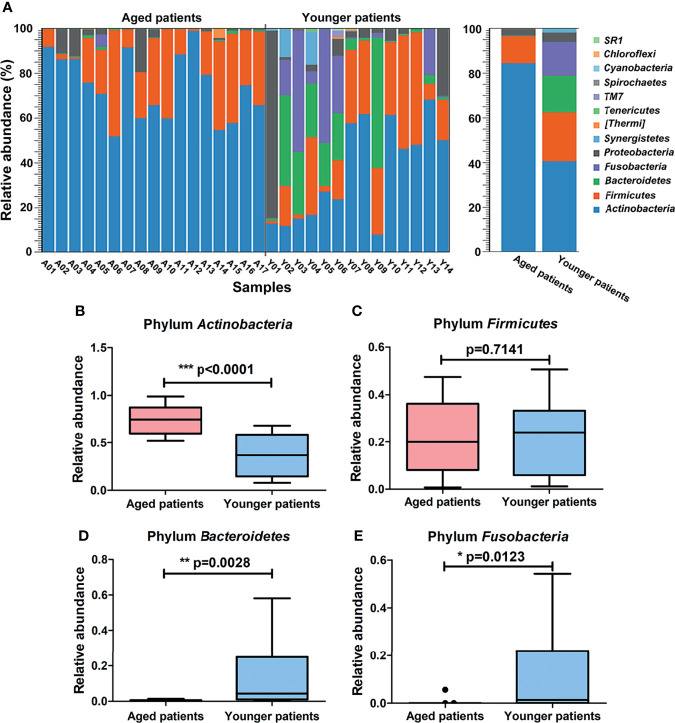
Differences in composition of bacterial community at the phylum level between aged and younger patients. **(A)** Relative abundance (%) of bacterial taxa at phylum level of the nasal cavity of patients with CRS and comparison of bacterial abundance between aged and younger patients. **(B–E)** Mean *Actinobacteria*, *Firmicutes*, *Bacteroidetes*, and *Fusobacteria* abundance were compared between aged and younger patients. *p < 0.05, **p < 0.01, and ***p < 0.001.

**Figure 4:**
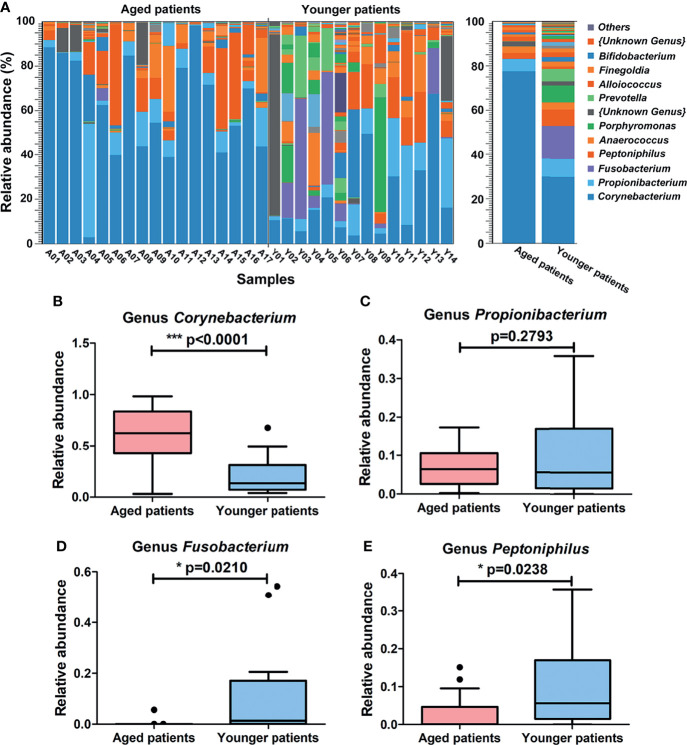
Differences in composition of bacterial community at the genus level between aged and younger patients. **(A)** Relative abundance (%) of bacterial taxa at genus level of the nasal cavity of patients with CRS and comparison of bacterial abundance between aged and younger patients. **(B–E)** Mean *Corynebacterium*, *Propionibacterium*, *Fusobacterium* and *Peptoniphilus* abundance were compared between aged and younger patients. *p < 0.05, and ***p < 0.001.

The abundance of annotated bacterial taxa and respective 16S rDNA gene sequences based on OTUs for aged and younger patients with CRS are shown in **Supplementary Table 2**. The bacterial community composition and differences in their relative abundance at the phylum and genus levels are visualized in **Figures 3** and **4**. At the phylum level, A total of 13 phyla were identified in the sequencing analysis, among which *Actinobacteria* were the most abundant phyla in both aged and younger patients, followed by *Firmicutes*, *Bacteroidetes*, and *Fusobacteria*, accounting for above 90% of all OTUs (**Figure 3A**). In aged patients, *Actinobacteria* was the dominant bacteria with a relative abundance of 84% (**Figure 3A**), which is significantly higher than that in younger patients (p < 0.0001, **Figure 3B**). The abundance of *Firmicutes* showed no significant difference in aged and younger patients (p = 0.7141, **Figure 3C**) while *Bacteroidetes*, and *Fusobacteria* were highly abundant in younger patients and significantly higher than that in aged patients (all p <0.05, **Figures 3D, E**).

At the genus level, a total of 107 bacteria genera were identified, among which *Corynebacterium* (77%) and *Propionibacterium* (6%) were the major genus in aged patients while *Corynebacterium* (30%), *Fusobacterium* (15%), *Propionibacterium* (8%), and *Peptoniphilus* (7%) were prevalent in younger patients (**Figure 4A**). When comparing the two groups, *Corynebacterium* showed a significantly higher relative abundance in aged patients than that in younger patients (p < 0.0001, **Figure 4B**). No significant difference of *Propionibacterium* was observed between aged and younger patients (p = 0.2793, **Figure 4C**) while *Fusobacterium* and *Peptoniphilus* showed a significantly higher relative abundance in younger patients than that in aged patients (all p < 0.05, **Figures 4D, E**).

### Difference in Predictive Functional Profiling of the Bacterial Community Between Aged and Younger Patients

**Figure 5:**
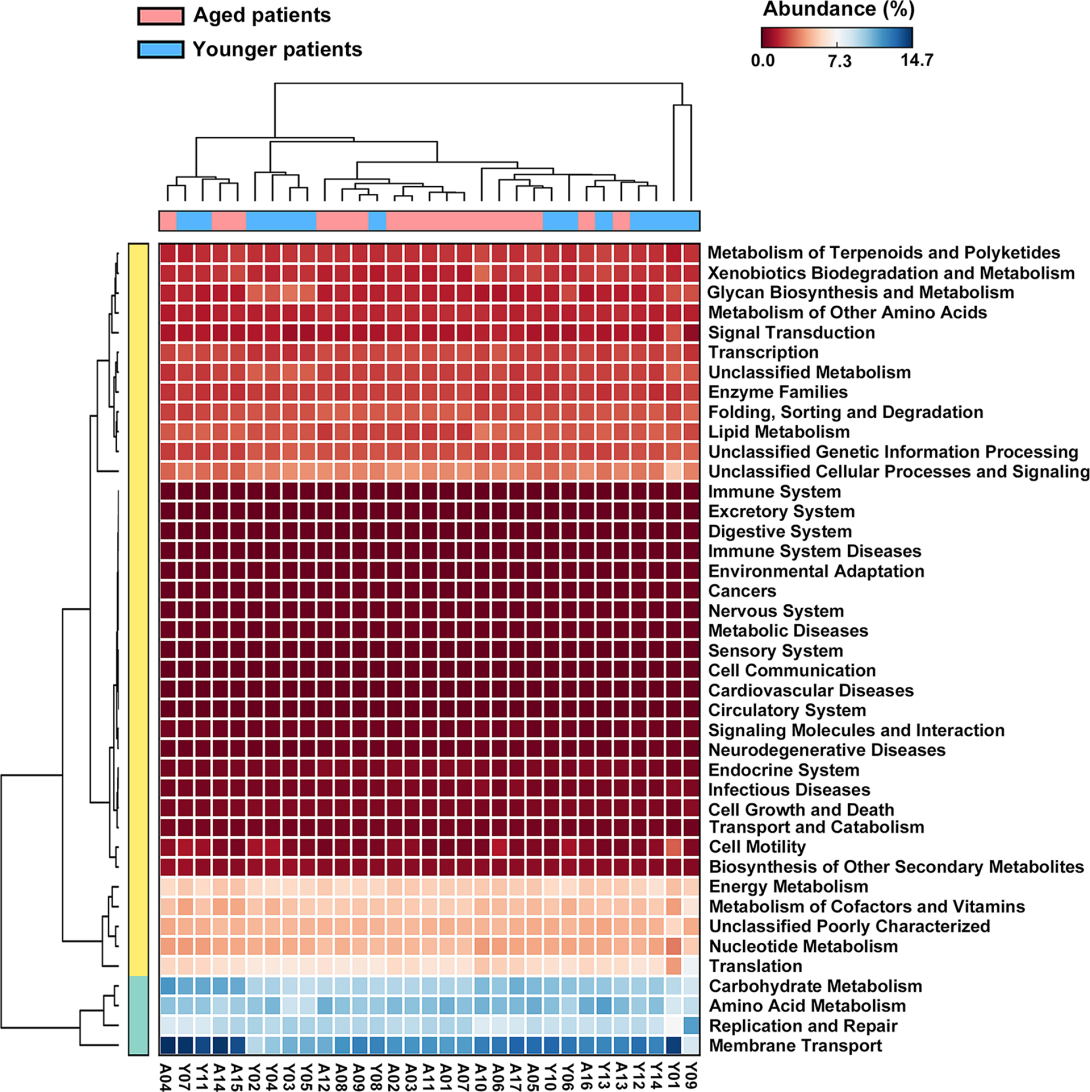
Heat map plot of bacterial functional features of the bacterial community in aged and younger patients as annotated using KEGG pathways.

**Figure 6:**
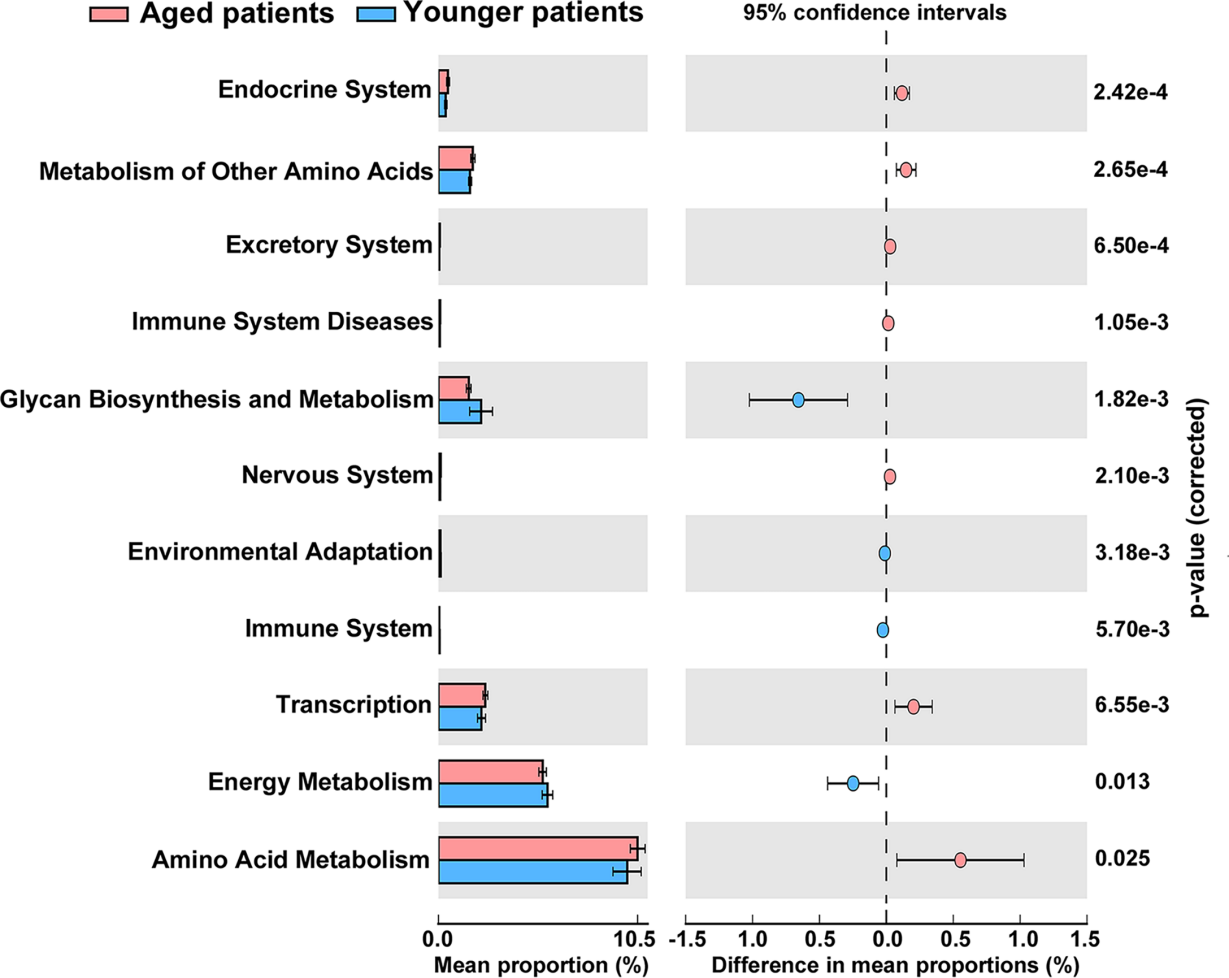
Significant differences in abundance of functional properties of the bacterial community regarding the super pathways between aged and younger patients.

**Figure 7:**
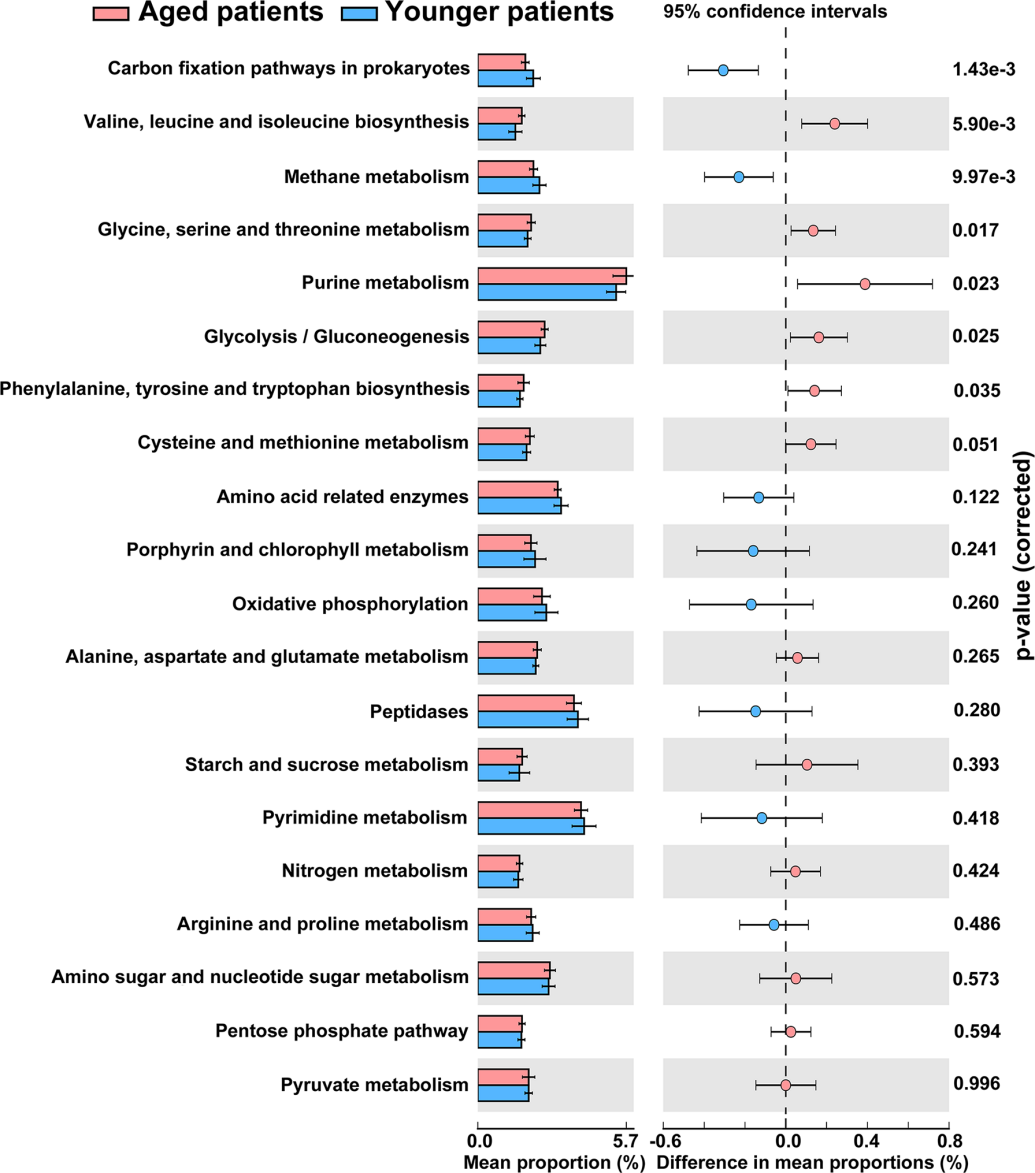
Differences in abundance of the thirdly bacterial metabolic pathways (the top 20 enriched) between aged and younger patients.

The metagenome functions of the bacterial community between aged and younger patients were predicted using PICRUSt2 (Douglas et al., 2020). Predicted functional profiles based on KEGG database were categorized into three levels: category, super pathway and sub pathway (**Supplementary Table 3**). According to mean proportion, most dominant predictive functionality regarding the categories in aged patients was metabolism (49.02%) followed by genetic information processing (20.38%), environmental information processing (14.15%), cellular processes (1.41%), human diseases (0.95%), organismal systems (0.90%), and unclassified (13.19% %), and similar results are also observed in younger patients (**Supplementary Figure 1**). **Figure 5** shows the heat map plot of specific enriched functional features of the bacterial community regarding the super pathways in aged and younger patients. It can be seen that 41 KEGG pathways involving 12 metabolic pathways, 4 genetic information processing pathways, 3 environmental information processing pathways, 4 cellular processes pathways, 8 organismal systems pathways, 6 human diseases pathways, and 4 unclassified pathways were identified. Among which, carbohydrate metabolism and amino acid metabolism, replication and repair and translation, and membrane transport were the main enriched pathways of the bacterial community both in aged and younger patients, belonging to metabolic pathways, genetic information processing, and environmental information processing, respectively. The vast majority of metabolic activities are involved in the metabolism of amino acid, carbohydrate, energy, cofactors and vitamins, and nucleotide, which correspond to the basic metabolism of microbes. The lipid metabolism, enzyme families, metabolism of terpenoids and polyketides, xenobiotics biodegradation and metabolism, and glycan biosynthesis and metabolism present higher portion of metabolic activity as well. When comparing the two groups, amino acid metabolism (p = 0.025), metabolism of other amino acids (p = 0.000265), endocrine system (p = 0.000242), excretory system (p = 0.00065), nervous system (p = 0.0021), immune system diseases (p = 0.00105), and transcription (p = 0.00655) showed a significantly higher abundance in aged patients than those in younger patients, while energy metabolism (p = 0.013), glycan biosynthesis and metabolism (p = 0.00182), environmental adaptation (p = 0.00318), and immune system (p = 0.0057) showed a significantly lower abundance in aged patients than those in younger patients (**Figure 6**). In addition, on the level of the thirdly bacterial metabolic pathways (sub pathways), purine metabolism (p = 0.023), glycine, serine and threonine metabolism (p = 0.017), valine, leucine and isoleucine biosynthesis (p = 0.0059), glycolysis/gluconeogenesis (p = 0.025) and phenylalanine, tyrosine and tryptophan biosynthesis (p = 0.035) are significantly up-regulated, while carbon fixation pathways in prokaryotes (p = 70.00143), and methane metabolism (p = 0.00997) are significantly down-regulated in aged patients when compared to younger patients (**Figure 7**).

### Correlations Between the Bacterial Diversity and Metabolic Pathways

**Figure 8:**
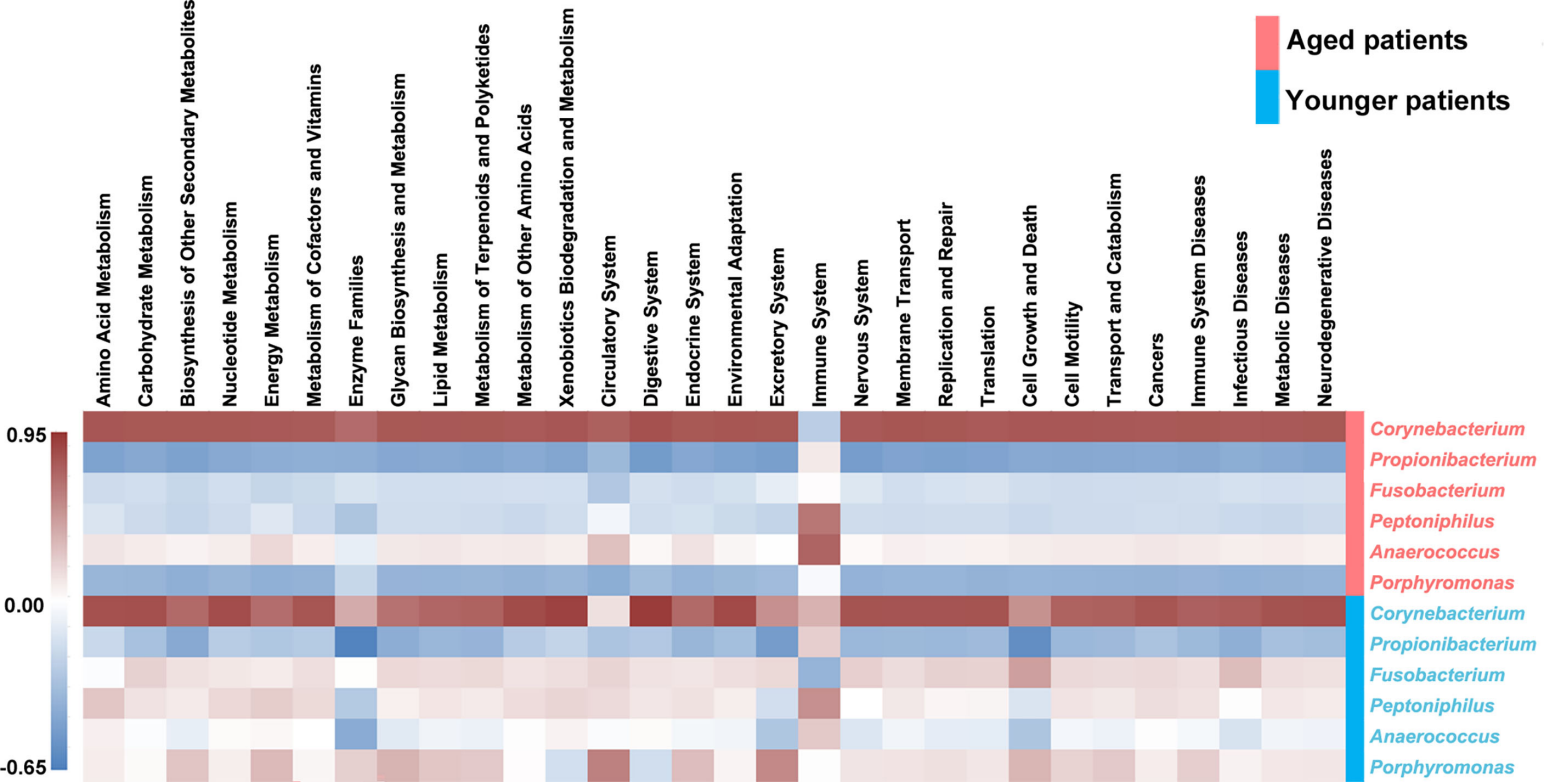
Heat map of the correlations between the differential abundant genus and the top 30 abundant secondary metabolic pathways. The Pearson correlation coefficient between the differential genus and the metabolic pathway was calculated, and the correlation coefficient matrix of the differential genus-metabolic pathway was obtained, and the matrix heat map was drawing by R language. Red represents a positive correlation, blue represents a negative correlation, and the darker the color, the greater the correlation.

In order to reveal what major variations in terms of specific genus identified to create a significant impact on the bacterial metabolic activity, correlations between differential genus and the top 30 secondary metabolic pathways were analyzed, as shown in **Figure 8**. It was clearly seen that *Corynebacterium* in aged patients was negatively associated with level of immune system while was positively related to level of immune system in younger patients. *Propionibacterium* was negatively correlated with levels of all the secondary metabolic pathways except for immune system both in aged patients and younger patients. *Fusobacterium* in aged patients showed negatively relationships with levels of all the secondary metabolic pathways except for immune system, while those in younger patients showed opposite correlations. In addition, opposite correlations of *Peptoniphilus, Anaerococcus* and *Porphyromonas* with metabolic pathways were also found in aged patients and younger patients.

## Discussion

A large number of studies have attempted to identify patient variables that drive CRS patterns and response to treatment (Jurlin et al., 2019; Morse et al., 2019; De Filippo et al., 2021; Soler et al., 2021). However, limited evidence suggests that advanced age can result in a unique CRS pathophysiology and potentially influences response to medical therapy. The role of aging in CRS pathophysiology remains poorly defined despite effects of age-related changes in immune function on airway inflammation have been extensively investigated Busse and Mathur, (2010). Age-dependent deterioration of the immune system may result in reduced phagocytosis of pathogenic bacteria and impaired innate defense mechanisms (Shaw et al., 2013). We hypothesized that aging in patients with CRS might be associated with unique nasal microbial diversity signatures. In the present study, 17 aged patients and 14 younger patients with no significant differences in demographically assessed variables, including sex, frequency of nasal polyps, disease duration, and SNOT-22 score, were enrolled to investigate the age-related changes in bacterial community of nasal cavity. Our main findings showed a decreased bacterial richness and diversity in aged patients with CRS compared to younger patients. The alpha-diversity values of aged patients became significantly lower than those of younger patients. Concerning the beta-diversity, we again observed that nasal microbiota of aged patients showed a greatly different bacterial community composition to the microbiota of younger patients. All these data suggest that age played an important role in developing nasal microbiota of patients with CRS. However, it is still unclear whether age influences nasal bacterial community composition in patients with CRS as the studies on nasal microbiota in relation to aging are very limited.

According to the work conducted by Lin et al. (2020), the change in microbiota could be beneficial if it is associated with a protective effect against allergic/atopic diseases, but it could also be worrying if it leads to the colonization of antimicrobial resistant bacteria. Therefore, it would be very interesting to know how the microbial signature varied between aged and younger patients with CRS even though the relationship between age and microbial signature is unclear. Our study identified several age-related specific CRS microbiomes. At the phylum level, *Actinobacteria* and *Firmicutes* were the two dominating phyla in aged patients while *Actinobacteria*, *Firmicutes*, *Bacteroidetes*, and *Fusobacteria* were the major dominant phyla in younger patients. These phyla include many organisms have been widely proved and commonly associated with CRS in previous research (Gan et al., 2021). For example, *Actinobacteria* includes *Corynebacterium* and *Firmicutes* includes *Staphylococcus* species, which are the most prevalent microbiotypes in sinonasal microbiome (Bassiouni et al., 2020) and the dominant species in patients with CRS (Jain et al., 2018). Remarkably, *Actinobacteria* in aged patients revealed significantly higher abundance than that in younger patients, which may be conducive to the improvement of nasal sinus mucosal inflammation, thereby reducing the recurrence of nasal polyps after surgery (Gan et al., 2021). We also found that *Bacteroidetes* and *Fusobacteria* in aged patients were significantly lower than that in younger patients. A study conducted by Kuhar et al. (Kuhar et al., 2018) indicated that the higher the relative abundance of the *Bacteroidetes* phylum in nasal microbiota of CRS patients, the more increased severity of inflammatory degree and the more obvious mucosal ulceration. Combined with our data and the results of these studies, we conclude that changes in bacterial community at phylum level between aged and younger patients are correlated with age-related nasal sinus mucosal inflammation and mucosal ulceration, which could help lead to new individual treatments or diagnostic biomarkers for patients with CRS. Larger trials are needed to confirm this conclusion.

At the genus level, *Corynebacterium* and *Propionibacterium* were the dominant bacterial genera both in aged and younger patients. *Corynebacterium* and *Propionibacterium* species both belong to *Actinobacteria*, which are often clinically regarded as normal flora when identified on culture and often comprising the most abundant genus represented in both CRS and control populations. A meta-analysis of the sinonasal microbiota in CRS revealed that increased abundance of *Corynebacterium* was associated with CRS and increased abundance of *Propionibacterium* could differentiate healthy sinuses from chronically inflamed sinuses by combining and reanalysing published bacterial 16S rRNA sequence data (Wagner Mackenzie et al., 2017). Based on the co-occurrence network inference (CoNet), linear discriminant analysis effect size (LDA-LEfSE) and similarity percentage (SIMPER) analysis, *Corynebacterium* was identified as a potential biomarker of CRS-associated sinonasal microbiota and *Propionibacterium* was identified as a gatekeeper, whose presence may be important for stabilizing the healthy bacterial community (Wagner Mackenzie et al., 2017). In a study conducted by Kim et al. (2020), CRSwNPs patients with optimal outcomes showed more enriched *Corynebacterium* at the genus level than those with suboptimal outcomes and less enriched than controls. In a previous study, patients with CRS without allergic rhinitis had lower relative abundance of *Corynebacterium* than those with allergic rhinitis, whereas had similar levels of *Corynebacterium* compared with control subjects, suggesting that the reduction in *Corynebacterium* species might reflect a type 2 inflammation environment more than CRS disease (Mahdavinia et al., 2018). All these indicate that *Corynebacterium* possesses a potential protective role in maintaining the health of the sinus mucosa. Our variance analysis showed that the relative abundance of *Corynebacterium* in aged patients was significantly higher than that in younger patients while no significantly difference in the relative abundance of *Propionibacterium* was observed, which indicated that age-related dysbiosis of *Corynebacterium* might be a potential indicator to provide personalized management of elderly patients with CRS.

In our results, the relative abundance of *Fusobacterium* and *Peptoniphilus* also showed a significantly difference between aged and younger patients. The relative abundance in aged patients was significantly lower than that in younger patients. As an opportunistic pathogen, *Fusobacterium* species are excellent biofilm formers and can have a symbiotic relationship with other microbial community members or its hosts, which may cause opportunistic infections (Brennan and Garrett, 2019). *Peptoniphilus* belongs to gram-positive anaerobic cocci isolated from human clinical infections and was able to utilize peptone as a sole carbon source (Citron et al., 2012). *Peptoniphilus* has been isolated in a large variety of human endogeneous polymicrobial infections, which involving members of the contiguous microbiota through contamination of initially sterile anatomical sites (Enault et al., 2020). Therefore, it can be inferred that higher abundance of *Fusobacterium* and *Peptoniphilus* may result in higher incidence risk of upper respiratory tract infection.

Our study further investigated the predictive functional profiling of the bacterial community based on KEGG database between aged and younger patients to provide insight into the metabolic activities of nasal microbiota. The abundance of predictive functional properties on the level of super pathway in aged patients and younger patients showed a similar distribution pattern, which is consistent with the dominant microorganisms identified. Significant differences in abundance of amino acid metabolism, energy metabolism, metabolism of other amino acids, glycan biosynthesis and metabolism, environmental adaptation, endocrine system, excretory system, nervous system, immune system, immune system diseases, and transcription were found between aged and younger patients. For the metabolic activities, our results have shown that the metabolic capabilities of aged patients that involved in the metabolism of purine, glycine, serine and threonine, valine, leucine and isoleucine biosynthesis, glycolysis/gluconeogenesis, and phenylalanine, tyrosine and tryptophan biosynthesis are stronger while carbon fixation pathways in prokaryotes and methane metabolism are declined to a certain extent when compared to younger patients. Among which, glycerine, serine, and threonine metabolism pathways represent the branches of glycolysis/gluconeogenesis, which have been described as important processes to help maintain the redox balance and energy levels in stress conditions by linking nitrogen and carbon metabolisms (Igamberdiev and Kleczkowski, 2018). To reveal the reasons for the variations in the metabolic processes between aged and younger patients, associations between differential genus and metabolic pathways were analyzed. Our results have shown that *Corynebacterium* in aged patients was negatively associated with level of immune system while was positively related to level of immune system in younger patients. In addition, opposite correlations of *Fusobacterium*, *Peptoniphilus, Anaerococcus* and *Porphyromonas* with the main metabolic pathways were observed in aged and younger patients. These data suggested that variations in terms of *Corynebacterium*, *Fusobacterium* and *Peptoniphilus* between aged and younger patients created significant impacts on the immune system and other bacterial metabolic activities. The changes in the levels of all metabolic pathways above are the results of sequencing data predictions, and the true levels of metabolites *in vivo* still need to be verified by subsequent experiments.

Given the significant differences in bacterial composition and bacterial metabolic activities between aged and younger patients, we speculate that age-related physiological and pathological changes on the nasal mucosal surface may alter the host immune response and be highly associated with the nasal bacterial microbiota of patients with CRS. However, the causal relationship still needs to be elucidated by more studies. Further investigations are also required to determine the impact of nasal mucosal surface bacterial niches on the development of CRS. There are several limitations in this research. First, the small and unequal sample sizes of aged patients and younger patients may affect the reliability of the results to make definitive conclusions. Second, the variation of specimens’ sites and the rapid turnover of mucus covering respiratory epithelium are confounding factors to sampling of the nasal microbiome. In addition, all analyses in the current study were conducted based on cross-sectional data. Substantial variability in the native microbiome between individuals makes it difficult to unravel differences in microbial community profiles. Furthermore, mechanisms of host-microbiota interactions are lacked. Nevertheless, our study presents new and interesting findings to the comparative study of nasal microbiome between aged and younger patients with CRS.

## Conclusions

This study attempted to reveal the changes of bacterial community between aged and younger patients with CRS. Significant differences were found in diversity, microbiota composition and metabolic activities of nasal microbiota between aged and younger patients. Microbiota diversity in aged patients decreased significantly compared to younger patients. The relative abundance of phylum *Actinobacteria* and genus *Corynebacterium* in aged patients was significantly higher than that in younger patients, which had an improving effect on the nasal sinus mucosa infammation and might reducing the recurrence of nasal polyps after surgery. In contrast, the relative abundance of phylum *Bacteroidetes* and genus *Fusobacterium* in aged patients was significantly lower than that in younger patients, indicating the more increased severity of inflammatory degree and the more obvious mucosal ulceration may be observed in younger patients. Besides, predicted functional profiles have revealed that aged patients have stronger metabolic activities involved in amino acid metabolism while have weaker metabolic activities involved in energy metabolism and glycan biosynthesis and metabolism. Age-related physiological and pathological changes on the nasal mucosal surface may be highly associated with the bacterial community in nasal cavity of patients with CRS. Future studies are needed to elucidate the causal relationship.

## Data Availability Statement

The original contributions presented in the study are included in the article/**Supplementary Material**. Further inquiries can be directed to the corresponding author.

## Ethics Statement

The studies involving human participants were reviewed and approved by The Institutional Review Board of Jinan University Guangzhou Red Cross Hospital. The patients/participants provided their written informed consent to participate in this study.

## Author Contributions

JS, YL, and FC conceived and designed the experiments. FC, WG, MX, and JJL performed the experiments. FC, WG, CY, JZL, and FY analyzed the data. FC wrote the manuscript. JS, YL, FC, JZL, and FY reviewed the manuscript. All authors contributed to the article and approved the submitted version.

## Funding

This work was supported by grants from National Outstanding Youth Fund Project of National Natural Science Foundation of China (81300814) and Health Science and Technology Project of Guangzhou (20211A010014).

## Conflict of Interest

The authors declare that the research was conducted in the absence of any commercial or financial relationships that could be construed as a potential conflict of interest.

## Publisher’s Note

All claims expressed in this article are solely those of the authors and do not necessarily represent those of their affiliated organizations, or those of the publisher, the editors and the reviewers. Any product that may be evaluated in this article, or claim that may be made by its manufacturer, is not guaranteed or endorsed by the publisher.

## References

[B1] Bassiouni A. Paramasivan S. Shiffer A. Dillon M. R. Cope E. K. Cooksley C.. (2020). Microbiotyping the Sinonasal Microbiome. Front. Cell. Infect. Microbiol. 10 (137). doi: 10.3389/fcimb.2020.00137 PMC7156599 32322561

[B2] Brennan C. A. Garrett W. S. (2019). Fusobacterium Nucleatum - Symbiont, Opportunist and Oncobacterium. Nat. Rev. Microbiol. 17 (3), 156–166. doi: 10.1038/s41579-018-0129-6 30546113 PMC6589823

[B3] Busse P. J. Mathur S. K. (2010). Age-Related Changes in Immune Function: Effect on Airway Inflammation. J. Allergy Clin. Immunol. 126 (4), 690–699. doi: 10.1016/j.jaci.2010.08.011 20920759 PMC3297963

[B4] Caporaso J. G. Kuczynski J. Stombaugh J. Bittinger K. Bushman F. D. Costello E. K.. (2010). QIIME Allows Analysis of High-Throughput Community Sequencing Data. Nat. Methods 7 (5), 335–336. doi: 10.1038/nmeth.f.303 20383131 PMC3156573

[B5] Cho S.-W. Kim D.-Y. Choi S. Won S. Kang H.-R. Yi H. (2021). Microbiome Profiling of Uncinate Tissue and Nasal Polyps in Patients With Chronic Rhinosinusitis Using Swab and Tissue Biopsy. PloS One 16 (4), e0249688. doi: 10.1371/journal.pone.0249688 33831071 PMC8031401

[B6] Cho S. H. Kim D. W. Lee S. H. Kolliputi N. Hong S. J. Suh L.. (2015). Age-Related Increased Prevalence of Asthma and Nasal Polyps in Chronic Rhinosinusitis and its Association With Altered IL-6 Trans-Signaling. Am. J. Respir. Cell Mol. Biol. 53 (5), 601–606. doi: 10.1165/rcmb.2015-0207RC 26266960 PMC4742956

[B7] Citron D. M. Tyrrell K. L. Goldstein E. J. C. (2012). Peptoniphilus Coxii Sp Nov and Peptoniphilus Tyrrelliae Sp Nov Isolated From Human Clinical Infections. Anaerobe 18 (2), 244–248. doi: 10.1016/j.anaerobe.2011.11.008 22178538

[B8] Cope E. K. Goldberg A. N. Pletcher S. D. Lynch S. V. (2017). Compositionally and Functionally Distinct Sinus Microbiota in Chronic Rhinosinusitis Patients Have Immunological and Clinically Divergent Consequences. Microbiome 5, 53. doi: 10.1186/s40168-017-0266-6 28494786 PMC5427582

[B9] DeConde A. S. Soler Z. M. (2016). Chronic Rhinosinusitis: Epidemiology and Burden of Disease. Am. J. Rhinol. Allergy 30 (2), 134–139. doi: 10.2500/ajra.2016.30.4297 26980394

[B10] De Filippo M. Votto M. Licari A. Pagella F. Benazzo M. Ciprandi G.. (2021). Novel Therapeutic Approaches Targeting Endotypes of Severe Airway Disease. Expert Rev. Respir. Med. 15 (10), 1303–1316. doi: 10.1080/17476348.2021.1937132 34056983

[B11] Delliere S. Dannaoui E. Fieux M. Bonfils P. Gricourt G. Demontant V.. (2021). Analysis of Microbiota and Mycobiota in Fungal Ball Rhinosinusitis: Specific Interaction Between Aspergillus Fumigatus and Haemophilus Influenza? J. Fungi 7 (7), 550. doi: 10.3390/jof7070550 PMC8305266 34356929

[B12] Douglas G. M. Maffei V. J. Zaneveld J. R. Yurgel S. N. Brown J. R. Taylor C. M.. (2020). PICRUSt2 for Prediction of Metagenome Functions. Nat. Biotechnol. 38 (6), 685–688. doi: 10.1038/s41587-020-0548-6 32483366 PMC7365738

[B13] Edgar R. C. (2010). Search and Clustering Orders of Magnitude Faster Than BLAST. Bioinformatics 26 (19), 2460–2461. doi: 10.1093/bioinformatics/btq461 20709691

[B14] Edgar R. C. (2013). UPARSE: Highly Accurate OTU Sequences From Microbial Amplicon Reads. Nat. Methods 10 (10), 996–9+. doi: 10.1038/nmeth.2604 23955772

[B15] Enault C. Aujoulat F. Pantel A. Cellier N. Lechiche C. Mégy B.. (2020). Surgical Site Infection After Hip Replacement Due to a Novel Peptoniphilus Species, Provisionally Named ‘Peptoniphilus Nemausus’ Sp. Nov. Anaerobe 61, 102071. doi: 10.1016/j.anaerobe.2019.102071 31306754

[B16] Eschenbacher W. Eid R. Borish L. (2021). Recent Discoveries Regarding the Pathogenesis of Chronic Rhinosinusitis and Their Implications for Future Therapies. Ann. Allergy Asthma Immunol. 126 (2), 107–108. doi: 10.1016/j.anai.2020.11.006 33509376

[B17] Fokkens W. J. Lund V. J. Hopkins C. Hellings P. W. Kern R. Reitsma S.. (2020). European Position Paper on Rhinosinusitis and Nasal Polyps 2020. Rhinology 58 29), 1–464. doi: 10.4193/Rhin20.600 32077450

[B18] Gan W. Zhang H. Yang F. Liu S. Liu F. Meng J. (2021). The Influence of Nasal Bacterial Microbiome Diversity on the Pathogenesis and Prognosis of Chronic Rhinosinusitis Patients With Polyps. Eur. Arch. Oto-Rhino-Laryngol. 278 (4), 1075–1088. doi: 10.1007/s00405-020-06370-4 32960349

[B19] Hayes S. M. Biggs T. C. Goldie S. P. Harries P. G. Walls A. F. Allan R. N.. (2020). Staphylococcus Aureus Internalization in Mast Cells in Nasal Polyps: Characterization of Interactions and Potential Mechanisms. J. Allergy Clin. Immunol. 145 (1), 147–159. doi: 10.1016/j.jaci.2019.06.013 31254531

[B20] Ho J. C. Chan K. N. Hu W. H. Lam W. K. Zheng L. Tipoe G. L.. (2001). The Effect of Aging on Nasal Mucociliary Clearance, Beat Frequency, and Ultrastructure of Respiratory Cilia. Am. J. Respir. Crit. Care Med. 163 (4), 983–988. doi: 10.1164/ajrccm.163.4.9909121 11282777

[B21] Igamberdiev A. U. Kleczkowski L. A. (2018). The Glycerate and Phosphorylated Pathways of Serine Synthesis in Plants: The Branches of Plant Glycolysis Linking Carbon and Nitrogen Metabolism. Front. Plant Sci. 9, 318. doi: 10.3389/fpls.2018.00318 PMC5861185 29593770

[B22] Jain R. Hoggard M. Zoing M. Jiang Y. Biswas K. Taylor M. W.. (2018). The Effect of Medical Treatments on the Bacterial Microbiome in Patients With Chronic Rhinosinusitis: A Pilot Study. Int. Forum Allergy Rhinol. 8 (8), 890–899. doi: 10.1002/alr.22110 29517178

[B23] Jang D. W. Lee H.-J. Huang R. J. Cheng J. Abi Hachem R. Scales C. D. (2021). Healthcare Resource Utilization for Chronic Rhinosinusitis in Older Adults. Healthcare 9 (7), 796. doi: 10.3390/healthcare9070796 34201975 PMC8305990

[B24] Jurlin L. Greguric T. Baudoin T. Grgic M. V. Pazanin L. Kosec A.. (2019). Cluster Analysis of Chronic Rhinosinusitis Suggests Gender-Based Differences. Orl-J. Oto-Rhino-Laryngol. Head Neck Surg. 81 (1), 1–9. doi: 10.1159/000492966 30458446

[B25] Kim J. H. Kim S. H. Lim J. Y. Kim D. Jeong I. S. Lee D. K.. (2020). Association Between the Sinus Microbiota With Eosinophilic Inflammation and Prognosis in Chronic Rhinosinusitis With Nasal Polyps. Exp. Mol. Med. 52 (6), 978–987. doi: 10.1038/s12276-020-0458-1 32595207 PMC7338545

[B26] Kim Y. S. Kim N. H. Seong S. Y. Kim K. R. Lee G.-B. Kim K.-S. (2011). Prevalence and Risk Factors of Chronic Rhinosinusitis in Korea. Am. J. Rhinol. Allergy 25 (3), E117–E121. doi: 10.2500/ajra.2011.25.3630 21679523

[B27] Kuhar H. N. Tajudeen B. A. Mahdavinia M. Heilingoetter A. Ganti A. Gattuso P.. (2018). Relative Abundance of Nasal Microbiota in Chronic Rhinosinusitis by Structured Histopathology. Int. Forum Allergy Rhinol. 8 (12), 1430–1437. doi: 10.1002/alr.22192 30240151

[B28] Lin X. Ren X. Xiao X. Yang Z. Yao S. Wong G. W. K.. (2020). Important Role of Immunological Responses to Environmental Exposure in the Development of Allergic Asthma. Allergy Asthma Immunol. Res. 12 (6), 934–948. doi: 10.4168/aair.2020.12.6.934 32935487 PMC7492518

[B29] Magoc T. Salzberg S. L. (2011). FLASH: Fast Length Adjustment of Short Reads to Improve Genome Assemblies. Bioinformatics 27 (21), 2957–2963. doi: 10.1093/bioinformatics/btr507 21903629 PMC3198573

[B30] Mahdavinia M. Engen P. A. LoSavio P. S. Naqib A. Khan R. J. Tobin M. C.. (2018). The Nasal Microbiome in Patients With Chronic Rhinosinusitis: Analyzing the Effects of Atopy and Bacterial Functional Pathways in 111 Patients. J. Allergy Clin. Immunol. 142 (1), 287–290. doi: 10.1016/j.jaci.2018.01.033 29452201 PMC6890201

[B31] Morse J. C. Li P. Ely K. A. Shilts M. H. Wannemuehler T. J. Huang L. C.. (2019). Chronic Rhinosinusitis in Elderly Patients is Associated With an Exaggerated Neutrophilic Proinflammatory Response to Pathogenic Bacteria. J. Allergy Clin. Immunol. 143 (3), 990–99+. doi: 10.1016/j.jaci.2018.10.056 30468775 PMC6408962

[B32] Orlandi R. R. Kingdom T. T. Hwang P. H. Smith T. L. Alt J. A. Baroody F. M.. (2016). International Consensus Statement on Allergy and Rhinology: Rhinosinusitis. Int. Forum Allergy Rhinol. 6 (S1), S22–S209. doi: 10.1002/alr.21695 26889651

[B33] Parks D. H. Tyson G. W. Hugenholtz P. Beiko R. G. (2014). STAMP: Statistical Analysis of Taxonomic and Functional Profiles. Bioinformatics 30 (21), 3123–3124. doi: 10.1093/bioinformatics/btu494 25061070 PMC4609014

[B34] Ramakrishnan V. R. Mace J. C. Soler Z. M. Smith T. L. (2017). Examination of High-Antibiotic Users in a Multi-Institutional Cohort of Chronic Rhinosinusitis Patients. Int. Forum Allergy Rhinol. 7 (4), 343–351. doi: 10.1002/alr.21903 28084683 PMC5386802

[B35] Shaw A. C. Goldstein D. R. Montgomery R. R. (2013). Age-Dependent Dysregulation of Innate Immunity. Nat. Rev. Immunol. 13 (12), 875–887. doi: 10.1038/nri3547 24157572 PMC4096436

[B36] Soler Z. M. Schlosser R. J. Bodner T. E. Alt J. A. Ramakrishnan V. R. Mattos J. L.. (2021). Endotyping Chronic Rhinosinusitis Based on Olfactory Cleft Mucus Biomarkers. J. Allergy Clin. Immunol. 147 (5), 1732–173+. doi: 10.1016/j.jaci.2021.01.021 33549569 PMC8113080

[B37] Stevens W. W. Lee R. J. Schleimer R. P. Cohen N. A. (2015). Chronic Rhinosinusitis Pathogenesis. J. Allergy Clin. Immunol. 136 (6), 1442–1453. doi: 10.1016/j.jaci.2015.10.009 26654193 PMC4680986

[B38] Stryjewska-Makuch G. Janik M. A. Klamińska-Cebula H. Kolebacz B. Ścierski W. Lisowska G. (2021). Bacteriological Analysis of Selected Phenotypes of Chronic Rhinosinusitis With Co-Existing Asthma, Allergy and Hypersensitivity to non-Steroidal Anti-Inflammatory Drugs. Adv. Dermatol. Allergol. Postępy Dermatol. i Alergol. 38 (1), 57–62. doi: 10.5114/ada.2021.104279 PMC8362779 34408567

[B39] Vickery T. W. Ramakrishnan V. R. Suh J. D. (2019). The Role of Staphylococcus Aureus in Patients With Chronic Sinusitis and Nasal Polyposis. Curr. Allergy Asthma Rep. 19 (4), 21. doi: 10.1007/s11882-019-0853-7 30859336 PMC6491045

[B40] Wagner Mackenzie B. Waite D. W. Hoggard M. Douglas R. G. Taylor M. W. Biswas K. (2017). Bacterial Community Collapse: A Meta-Analysis of the Sinonasal Microbiota in Chronic Rhinosinusitis. Environ. Microbiol. 19 (1), 381–392. doi: 10.1111/1462-2920.13632 27902866

[B41] Yancey K. L. Lowery A. S. Chandra R. K. Chowdhury N. I. Turner J. H. (2019). Advanced Age Adversely Affects Chronic Rhinosinusitis Surgical Outcomes. Int. Forum Allergy Rhinol. 9 (10), 1125–1134. doi: 10.1002/alr.22404 31454179 PMC6773466

