# Supplementary figures and images for: Age-Associated Changes of Nasal Bacterial Microbiome in Patients With Chronic Rhinosinusitis
Source: Front Cell Infect Microbiol. 2022 Feb 17;12:786481. doi: 10.3389/fcimb.2022.786481 (PMC8891534; doi:10.3389/fcimb.2022.786481)

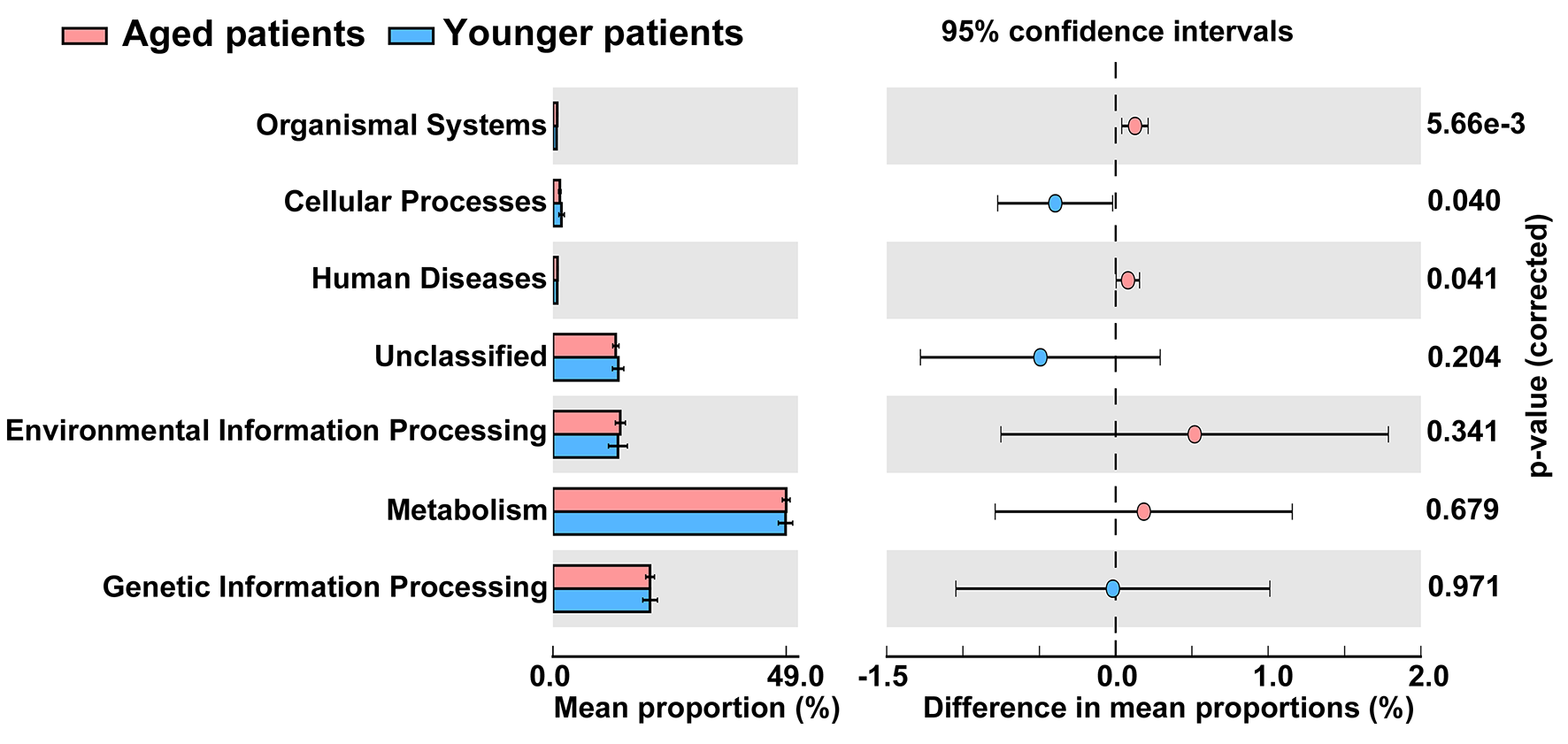

Supplement: Supplementary file 1 [file Image_1.tif]
