# Supplementary material for: Age-Associated Changes of Nasal Bacterial Microbiome in Patients With Chronic Rhinosinusitis
Source: Front Cell Infect Microbiol. 2022 Feb 17;12:786481. doi: 10.3389/fcimb.2022.786481 (PMC8891534; doi:10.3389/fcimb.2022.786481)
Supplement: Supplementary file 2 [file Table_1.docx]

**Table S1.**The information of sequences identified in aged and young patients with CRS.

|  | | **Read counts** | | | **Read length** | | | **Taxonomic assignment** | |
| --- | --- | --- | --- | --- | --- | --- | --- | --- | --- |
|  |  | **Raw PE reads** | **Valid reads** | **Effective Ratio (%)** | **Min** | **Max** | **Average** | **Reads in OTUs** | **OTUs** |
| **Aged patients** | **A01** | 62997 | 62556 | 99.3 | 330 | 470 | 452.7 | 9121 | 16 |
|  | **A02** | 85313 | 84631 | 99.2 | 320 | 476 | 447.6 | 6158 | 28 |
|  | **A03** | 70359 | 69937 | 99.4 | 309 | 477 | 448.1 | 4529 | 34 |
|  | **A04** | 60405 | 59919 | 99.2 | 370 | 477 | 448.3 | 914 | 56 |
|  | **A05** | 65417 | 65155 | 99.6 | 320 | 469 | 448.2 | 1284 | 45 |
|  | **A06** | 68821 | 68565 | 99.6 | 329 | 473 | 447.8 | 795 | 22 |
|  | **A07** | 52963 | 52718 | 99.5 | 320 | 474 | 458.4 | 4760 | 28 |
|  | **A08** | 66221 | 65978 | 99.6 | 364 | 474 | 448.0 | 787 | 16 |
|  | **A09** | 55105 | 54823 | 99.5 | 320 | 474 | 464.4 | 4148 | 47 |
|  | **A10** | 69746 | 69502 | 99.7 | 350 | 475 | 448.8 | 1397 | 27 |
|  | **A11** | 56478 | 56288 | 99.7 | 305 | 476 | 457.6 | 6595 | 40 |
|  | **A12** | 60528 | 60258 | 99.6 | 310 | 476 | 460.5 | 9621 | 30 |
|  | **A13** | 52211 | 52044 | 99.7 | 310 | 470 | 448.6 | 2014 | 29 |
|  | **A14** | 62705 | 62425 | 99.6 | 310 | 473 | 448.2 | 1165 | 40 |
|  | **A15** | 62036 | 61805 | 99.6 | 330 | 472 | 448.4 | 617 | 22 |
|  | **A16** | 75071 | 74736 | 99.6 | 324 | 472 | 447.6 | 2258 | 30 |
|  | **A17** | 63437 | 63105 | 99.5 | 320 | 471 | 458.1 | 1127 | 41 |
| **Younger patients** | **Y01** | 64883 | 64559 | 99.5 | 320 | 476 | 448.9 | 654 | 25 |
|  | **Y02** | 55561 | 55172 | 99.3 | 320 | 475 | 448.7 | 2304 | 42 |
|  | **Y03** | 68739 | 68023 | 99.0 | 333 | 470 | 447.8 | 2756 | 39 |
|  | **Y04** | 52192 | 51977 | 99.6 | 320 | 478 | 454.4 | 1338 | 40 |
|  | **Y05** | 48039 | 47858 | 99.6 | 360 | 470 | 448.0 | 2951 | 31 |
|  | **Y06** | 59179 | 58706 | 99.2 | 301 | 470 | 448.4 | 440 | 116 |
|  | **Y07** | 58938 | 58389 | 99.1 | 326 | 478 | 448.8 | 751 | 74 |
|  | **Y08** | 69018 | 68820 | 99.7 | 320 | 475 | 457.6 | 6515 | 56 |
|  | **Y09** | 76646 | 76314 | 99.6 | 382 | 476 | 448.5 | 3416 | 43 |
|  | **Y10** | 70616 | 70383 | 99.7 | 374 | 473 | 454.6 | 2047 | 56 |
|  | **Y11** | 62694 | 62500 | 99.7 | 312 | 476 | 450.2 | 942 | 35 |
|  | **Y12** | 58903 | 58306 | 99.0 | 310 | 477 | 448.4 | 1259 | 36 |
|  | **Y13** | 72352 | 72094 | 99.6 | 390 | 478 | 452.5 | 4851 | 48 |
|  | **Y14** | 60610 | 60435 | 99.7 | 326 | 477 | 448.6 | 454 | 36 |
